# The Present Outbreak of Smallpox in Buffalo1Read at the eighty-first annual meeting of the Medical Society of the County of Erie, January 14, 1902.

**Published:** 1902-02

**Authors:** Walter David Greene

**Affiliations:** Commissioner of Health of the City of Buffalo


### The Present Outbreak of Smallpox in Buffalo.[1]

BY WALTER DAVID GREENE, M.D.,
Commissioner of Health of the City of Buffalo.

THE first case of smallpox which was followed by the present outbreak of that disease, occurred on April 14, 1901, since which time Buffalo has not been entirely free from its presence. Before any individual case had entirely recovered another would occur, but not until November 20 did the disease become general and so far, with the exception of about 9 cases, has been confined to a district of our city, bounded by William, Jefferson and Sycamore Streets, and Walden and Bailey Avenues.

1. Read at the eighty-first annual meeting of the Medical Society of the County of Erie, January 14, 1902.

The difficulties encountered in handling smallpox have been greatly enhanced by the opposition of the inhabitants of the district who hamper the work of the health authorities in every possible way. Many of them strenuously object to having their children vaccinated and especial objection is made to vaccinating the heads of families, the reason given being that a sore arm precludes work.

In several instances living rooms have been found locked, and doors and windows guarded by a man with a club or axe in his hand, who threatened violence to the physician who sought entrance for the purpose of investigation and vaccination of the inmates. This self-appointed guardian has, in some instances, been assisted by his wife. Police aid is constantly required and the thanks of the health department is due and is hereby tendered the police authorities for their active coöperation.

Several factors combine to counteract the efforts put forth to stamp out the disease. One, more prominent than any other, is the disposition on the part of the inhabitants to conceal the fact when cases occur in any given family. The reason given is that the father, who is the breadwinner, must be quarantined should the case be discovered, or must remain away from his family from four to six weeks. Another, is the exceedingly mild form of the disease as it now exists. The great majority of cases require no treatment whatever. If it were of a more severe type a physician would be called to treat it, and discovery and quarantine would follow.

Several children have called at the vaccination station to be vaccinated who were recovering or had fully recovered from smallpox. Another prolific cause is the fact that we have no hospital worth the name for housing and quarantining those who suffer from this malady. In the so-called isolation hospital, there are two rooms in which the patients are cared for, something like the general ward in any of our city hospitals,—one for males and one for females. Women of all colors and nationalities are huddled together, their ages being from the old woman down to the nursing baby, the predominating number being children under fourteen years of age. This building is heated with stoves and lighted by kerosene oil lamps. The roof of one part of this building is in such a condition that wash-tubs have to be used to catch the water that pours through the openings during a rainstorm. Then, again, the fact is apparent that it is not large enough even if it was in proper sanitary condition.

Smallpox is not as contagious in its early as it is in its later stages, and if a patient is taken early to an isolation hospital, and the other members of the family given an antiseptic bath, vaccinated, their clothing and the house fumigated and disinfected with formaldehyde gas, the danger from spreading the disease is minimised to such an extent that no further steps are necessary. But, when the patient must remain at home, for want of a proper isolation hospital, the entire family must remain under quarantine for several weeks, and all must be fed at the city's expense.

In the section where it now exists, five families occupy the same house in many instances, the sum total inhabiting a single house being 25 to 30, and with only one closet. That condition means that, if a case of smallpox occurs in that house, the entire number must be quarantined and fed at the city's expense for from four to six weeks, or until desquamation has entirely ceased. In the meantime other cases are liable to occur in the same house, thus lengthening the time of quarantine and feeding to possibly three or four months. It also requires the services of three policemen for every twenty-four hours. I do not mean to convey the idea that this measure is adopted in every case, but when the hospital is filled, then the above condition, of necessity, must exist. The total number of cases discovered and reported from April 14, 1901, to January 14, 1902, has been 237; number treated at isolation hospital, 65; patients in hospital at present time, 30; other members of families in hospital, 8; houses under quarantine at present time, 34; families under quarantine at present time, 55; persons under quarantine at present time, 314; total number of persons under quarantine from October 12, 1901, to January 14, 1902, about 1,100; total deaths, 3.

The city was employing at one time about 160 policemen for quarantine duty, and feeding about 475 people outside of the hospital. What the cost will be will depend of course upon the length of time it will take to stamp out the disease. Six physicians are now regularly employed, making a house to house search for concealed cases and vaccinating the inmates. An accompanying policeman is necessary in every case. All the cases discovered for several days have been found by this exploring party, with the exception of those occurring in families where the disease already exists. The cost of the outbreak of smallpox in 1872 and 1873 was, in round numbers, $46,000, of which $24,000 was paid to physicians for their services in vaccinating. Fifty cases were reported in one day during that epidemic. The total number reported in the outbreak in 1889 was 147, with a total cost of about $22,000.

The mild character of smallpox attains all over the United States, which country never had so much smallpox in its history as at present.

From June 28, 1901, to December 6, 1901, smallpox was present in thirty-eight states and territories, Ohio leading with 3,466, then follows Minnesota with 2,245, Tennessee with 2,155, and Pennsylvania with 1,614, the grand total of cases occurring during that period in the United States being 15,549, with total deaths of 502, making a death-rate of about 3.22 per cent.

The reason of the mild character of smallpox throughout the country is not definitely known, but I suggest that it is largely due to the hereditary transmission of immunity. We all believe that the tendency to tuberculosis is transmitted from parent to child, and when we take into account the fact that the Anglo-Saxon race is a well vaccinated race, and has been for several generations, it seems rational to believe that the immunity may be hereditary.

315 Jersey Street.

